# Using Participatory Action Research to Develop a Working Model That Enhances Psychiatric Nurses’ Professionalism: The Architecture of Stability

**DOI:** 10.1007/s10488-017-0806-1

**Published:** 2017-05-18

**Authors:** Martin Salzmann-Erikson

**Affiliations:** Department of Health and Caring Sciences, Faculty of Health and Occupational Studies, University of Gävle, 801 76 Gävle, Sweden

**Keywords:** Action research, Ethics, Nursing, Mental health nursing, Psychiatric ward, Ward rules

## Abstract

Ward rules in psychiatric care aim to promote safety for both patients and staff. Simultaneously, ward rules are associated with increased patient violence, leading to neither a safe work environment nor a safe caring environment. Although ward rules are routinely used, few studies have explicitly accounted for their impact. To describe the process of a team development project considering ward rule issues, and to develop a working model to empower staff in their daily in-patient psychiatric nursing practices. The design of this study is explorative and descriptive. Participatory action research methodology was applied to understand ward rules. Data consists of audio-recorded group discussions, observations and field notes, together creating a data set of 556 text pages. More than 100 specific ward rules were identified. In this process, the word *rules* was relinquished in favor of adopting the term *principles*, since rules are inconsistent with a caring ideology. A linguistic transition led to the development of a framework embracing the (1) Principle of Safety, (2) Principle of Structure and (3) Principle of Interplay. The principles were linked to normative guidelines and applied ethical theories: deontology, consequentialism and ethics of care. The work model reminded staff about the principles, empowered their professional decision-making, decreased collegial conflicts because of increased acceptance for individual decisions, and, in general, improved well-being at work. Furthermore, the work model also empowered staff to find support for their decisions based on principles that are grounded in the ethics of totality.

## Introduction

Working conditions for staff in psychiatric inpatient care, with a special focus on psychiatric intensive care units, include interactions with patients with acute mental illness. In their daily work environments, staff are exposed to patients’ different forms of expression in their condition—an environment that is characterized by interactions with many different people, unpredictability, and the occurrence of hints of threats and violence (Salzmann-Erikson 2013). Working life varies on the basis of these conditions, making extensive demands on caregivers to maintain professional, ethical and caring approaches. This study is a 9-month-long participatory action research project conducted at a psychiatric intensive care unit in Sweden. The aims of the project were to describe the process of a team development project considering ward rule issues, and to develop a working model to empower staff in their daily in-patient psychiatric nursing practices. As exemplified in the methodology, the specific research focus was not predetermined, but was decided together with the staff during the problem identification and planning phase.

### Disciplining Practices to Make Patients ‘Behave Themselves’

Dating back in history, people who demonstrated deviant behaviors were kept away from public spaces in asylums and mental health institutions, where restrictions and limitations were practiced in various forms (Colaizzi 2005). In Goffman’s (1961) classic research, he described the socializing processes of inmates who had to submit to institutional rules. The first serious discussion and critical analysis of exercising control over patients in psychiatric wards emerged during the twentieth century with influential thinkers such as Erving Goffman, Michel Foucault, Ronald David Laing and Thomas Szasz, who addressed aspects of history, identity, power and socio-politics. In the wake of those thinkers, criticism of psychiatric nursing has been voiced (Crichton 1997; Holmes 2005). In search of better psychiatric care, several researchers have reported successful alternatives to coercion, such as adopting a person-centered approach (Georgieva et al. 2009; Mullen and Drinkwater 2011; Qurashi et al. 2010). Relics of such socializing processes of inmates who had to submit to institutional rules are still present in contemporary psychiatric care, through the practice of various ward rules, restrictions and coercive measures (Noorthoorn et al. 2015; Salzmann-Erikson 2014; Vatne and Holmes 2006). For example, it is common to lock the entrance doors of psychiatric inpatient wards in order to maintain control of who is inside and outside the ward, and to head off patients’ escape attempts (Haglund et al. 2006; Muir-Cochrane et al. 2012). Using seclusion and restraints is another formalized way of controlling patients’ externalizing behaviors and is justified in most countries’ legislation, including that of Sweden, the United Kingdom and Australia (see DoH 2012; MHA 2014; SFS 1991:1128). Previous studies have reported that staff use seclusion and mechanical restraint for up to 20% of in-patients (Beghi et al. 2013; Noorthoorn et al. 2015). However, policies stress that coercion is to not be practiced routinely but to be considered ‘the last resort’ (Mérineau-Côté and Morin 2013). Formal coercion is beyond the scope of this study. Instead, this study focuses on the limits staff set in wards.

Psychiatric staff use the term *limit-setting* to refer to an intervention that controls or prevents patients’ disruptive behaviors (Usher et al. 2005; Vatne and; Fagermoen 2007). However, limit-setting is also associated with risks, since it may evoke negative feelings among patients and hence increase the risk of inducing violence (Dubin and Ning 2008). As concluded by Maguire et al. (2014), setting limits is necessary in psychiatric wards to ensure well-being and safety. Howeverthe authors emphasize that *how* limits are set and presented to the patients will determine their responses.

More subtle ways of controlling patients also exist, particularly in terms of staff’s enforcement of ward rules and techniques to make patients ‘behave themselves’ (Hall 2004). The knowledge regarding these subtle forms of controlling patients is limited because of research methodological challenges. Ward rules and less direct forms of controlling patients have been accounted for in some qualitative studies, although these pose much broader research questions using ethnography or similar methodologies. Morrison (1990) conducted an ethnographic study in a forensic psychiatric ward, where he identified a “culture of toughness.” Morrison described that staff maintained a hegemonic position over patients by demonstrating authority and power and exerted a police-like role in order to control patients. Moreover, Watters (2000) published a study in which he described the process of socializing patients into the rules of the psychiatric hospital. Watters explained that staff used power strategies when patients refused medication: they withdrew privileges such as phone calls, visitors, and smoking, and increased confinement and medication. If patients did not conform to the routines, the staff’s task was to make the patients aware of the consequences. Similarly, Hall (2004) reported that staff viewed controlling patients as a way to “make sure that the patients are behaving themselves within an acceptable level” (p. 544).

Another ethnographic study conceptualized a framework for ‘keeping the unit safe’ and addressed four dimensions of the framework: ideology, space, time and people (Delaney and Johnson 2006). They described that ward rules were set up in order to fulfill the ideology of ensuring safety. For example, they reported that patients were prohibited from entering another patient’s room, that patients were not allowed to borrow or lend items to each other, nor were they allowed to have bodily contact. Other researchers have looked at similar phenomena under various terminology. In the practice of limit-setting, nurses used subtle coercion in order to take control, for example, by making deals with patients, in combination with rewards or loss of privileges (Vatne and Holmes 2006). In an ethnographic observational study conducted by Salzmann-Erikson (2014), it was reported that patients who violated ward rules and boundaries were first corrected in a nice, friendly manner by staff, but as the violations of boundaries continued, more hostile language was used. In one example, a staff member yelled, “What the hell are you doing in here, you are not allowed to be in here just so you know—do not do that again, you know you are not allowed to be there!!” (p. 247).

### Problems Associated With Rigid Nursing Teams and Ward Rules

There are several problems associated with rigid nursing approaches; for example, patients feel humiliated, confined and angry. Such negative aspects may also lead to a risk of increased turmoil throughout the ward, hence decreasing safety (Alexander 2006; Salzmann-Erikson 2013). Moreover, Crichton (1997) stated that *discipline* is a useful word for the asserted control that staff use on patients in their attempts to control ‘unacceptable behavior’. An interesting aspect that Crichton adds to the debate was that the effect of ward rules is not only to support staff that oppress patients; ward rules also “bind both staff and patients to behave in an accepted way” (p. 37).

In conclusion, few studies have explicitly accounted for the research question of ward rules. The available literature that has tangential findings on ward rules has predominantly focused on banned items from a safety perspective (Bowers et al. 2002; Koukia et al. 2010; Salzmann-Erikson 2014). Also, studies that have primarily accounted for patients’ experiences in acute psychiatric wards in general have reported that ward rules have an impact on their experience, mostly a negative one (Lilja and Hellzén 2008; Shattell et al. 2008; Skorpen et al. 2008). The practice of disciplining patients in psychiatric nursing has been criticised (Crichton 1997; Holmes 2005), and many studies have tried to develop working models that are more person-centered (Georgieva et al. 2009; Mullen and Drinkwater 2011; Qurashi et al. 2010). In contrast, Steinert, Kappenschneider and Flammer (2016) valued ward rules as an important element of treatment. Furthermore, reports of staff injuries from patient assault fuel the exhortation for a ‘zero tolerance’ policy, in order to establish safe workplaces for staff (Harder 2008; Joint Commission 2008; Tavernero 2009). Even though safe workplaces are important, several scholars have noted risks with the zero-tolerance policy. Among others, Whittington (2002) and Bower et al. (2009) have pointed out that zero-tolerance policies risk further entrenching a reduced general tolerance within a work group, and this may then spread to permeate the entire work culture. Consequently, the restrictions put on patients as a safety measure may actually exacerbate the problem of violence as a repercussion. Thus, the discourse does not indicate consensus.

Asserting control of patients through the use of, for example, ward rules, carries with it a inherent risk since patient violence is often associated with staff attitudes, withholdingprivileges (for example smoking), limit-setting and failure to interact with patients (Duxbury and Whittington 2005; Roper and Anderson 1991; Vatne and Holme 2006). In addition, it has been emphasized that nurses find controlling practices incongruent with their moral values and ideology of authentic nursing care, which may put moral stress on nurses and their working conditions (Alexander 2006; Lützén 1990). Alexander and Bowers (2004) concluded that there is a gap in the literature of studies explicitly addressing the issue of ward rules. Thus, the objectives in this study were to describe the process of a team development project considering ward rule issues, and to develop a working model to empower staff in their daily in-patient psychiatric nursing practices.

## Methodology

The design of the study was explorative and descriptive. A participatory action research (PAR) methodology was adopted. PAR is a research style or “an orientation to inquiry”, rather than a step-by-step research method (Reason and Bradbury 2008, p. 1). The present study was grounded in democracy, trust, mutuality and openness (see Bergold and Thomas 2012). The departure point of the methodology is an inquiry into concerns or problems in the social setting, and it is the researcher and participant who form the research questions. The research style is also described as educative, empowering, and development-oriented, and the PAR tenets are to collaborate with practitioners, implement change and improve practice (Bergold and Thomas 2012; Lewin 1946; Waterman et al. 2001). Moreover, PAR doctrine asserts that research and the social realm intersect, hence PAR is grounded in an ideographic epistemology. On equal terms, theory and practice help each other in a developing process (Brydon-Miller et al. 2003). Hence, PAR rejects objectivist assumptions that distance the researcher and the research object. Rather, the idea in this study was to merge the researcher within the social practice. Thus, participants were on some occasions collaborators in the process of gathering and interpreting data and served as co-researchers and analysts; in addition the author/researcher is termed “I” (Boylorn 2006; Chilisa 2012). Due to these interwoven roles, the process of data collection and data analysis has been viewed as cyclical and ongoing throughout the process, rather than a set of predetermined steps, as in nomothetic epistemology. The PAR process includes four different phases: problem identification, planning, action and reflecting (Elden and Levin 1991; Springett et al. 2005). The use of PAR within healthcare research is useful in collectively solving problems and to educate and empower staff (Soh et al. 2011). Furthermore, Soh et al. (2011) demonstrate that action research promotes effective communication, change of work culture, increased awareness related to the issue, and improved partnership in care among patients and other healthcare professionals. Prior to the study, the research project was reviewed by the Regional Ethical Review Board [number cloaked during review process], which found that there were no ethical barriers to conducting the research project.

### Researcher-As-Instrument Statement

The active role of the researcher in PAR acknowledges the view of researcher-as-instrument (Bergold and Thomas 2012; Hammersley and Atkinson 1995) From this viewpoint, methodologists such as Morrow (2005) and Padgett (2008) stress the importance of self-disclosure and researcher-as-instrument statements to maintain trustworthiness, thus this section.

My clinical experience of psychiatric nursing dates back to the early 21th century. In parallel with my clinical experience, I have conducted research within the field of psychiatric and mental health nursing for about 10 years. During my time as a PhD student, I gained experience of conducting field work in in-patient wards, though as an ethnographer. My philosophy of psychiatric and mental health nursing originates from the ethical assumption of human dignity, and from the wish to help patients to move forward in their recovery processes in support of a nursing commitment and conscience. During the process of this project, I used self-disclosure throughout the project, but to various extents in order to gain trust within the work group. The aspects of researcher-as-instrument and reflexivity are given further attention in the Discussion section.

### Participants

An initial meeting was arranged with the unit manager in order to discuss the research project. Next, the management group, including all middle and section managers, were given a verbal presentation on the research project. Thereafter, the unit manager and the section manager were provided with detailed written information and signed this to indicate their consent. The staff at the unit were informed on three occasions in order to reach as many of them as possible, due to scheduling difficulties. The staff expressed a positive attitude toward the research project and the PAR methodology. In addition to the verbal information, all staff members were given a written information sheet and signed this to indicate their informed consent. The staff consisted of one unit manager, 11 male staff (two of whom were registered nurses and nine psychiatric aides) and 14 women (eight of whom were registered nurses and six psychiatric aides). No further data were collected about the staff as individuals, but anecdotal information revealed that the majority had several years of experience of working in the unit or other in-patient wards.

The staff turnover was minimal during the project; only one staff member returned from sick leave and was then informed about the study. However, due to staff shortages there was often a need for hourly personnel. Physicians, social workers, students, hospital priests and hourly personnel were informed about the research project to various degrees, but not included as participants; they were also informed about their exclusion. One staff did not agree to participate, and no data were collected from that person. In the following portions of this article, participants will mainly be referred to as the staff or the work group; on some occasions, a participant will be referred to as *the nurse, the psychiatric aide*, or by a fictitious name. The manager will also be referred to as one of the nurses to avoid disclosure. Sometimes “we” is used, which then refers to the close collaboration between myself as a researcher and the staff.

### Setting

In Sweden, there are about 15 psychiatric intensive care units (approximately 130 beds) for a population of 9.8 million people. This study was carried out at one such psychiatric intensive care unit, chosen from a convenience sample. The unit offered nine individual rooms for patients in the most critical phases of mental illness. The environment in the unit was highly restricted. When entering the front door, there was a holding area, which was used to prevent escapes and also used for holding patients when searching for prohibited items before entering the unit. The unit had the shape of the letter L with some additional small spaces. In general, all doors were kept closed, and some were also locked. In the sitting-room, there were some chairs and sofas, and my general feeling was that the environment was minimalistic and clean, but could not be described as inviting or overly comfortable. The unit admitted patients based on their need for care, and was not restricted to specific diagnoses. The unit predominantly cared for patients who were involuntarily admitted under the Mental Health Act (SFS, 1991:1128). Though the unit allowed voluntary admissions, those patients were in the minority.

Staff levels at the unit on day and evening shifts were set as a minimum of one registered nurse and three psychiatric aides, including a minimum of two male staff on each shift. At night, two psychiatric aides were present at the unit, and the registered nurse was responsible for an additional ward. The morning shift was set from 06:45–15:30, the evening shift, 13:00–22:00 and the night shift, 21:00–07:00. Prior to each shift, the staff met to report, with a clear patient focus. Monday through Thursday afternoons were often scheduled as staff meetings, work environment meetings, ethics rounds and other staff meetings.

### Data Collection

The data collection and data analysis processes were simultaneous. Grounded in the philosophy of constructivism, I did not view ‘data’ to be out there as something that could be collected. Rather, from deep involvement and interaction with the staff over time, we constructed data by a process-driven accumulation of insights from reciprocal interpretations of reality (Charmaz 2006; Glaser 1978; von Glasersfeld 1984).

Several methods were used to construct data: participant observations (spending time in the ward milieu together with the staff, actively attending meetings, formal reports, and breaks), informal interviews (conversations and chats), documents, field notes and analytic memos, and to a minor extent, electronic mail correspondence. During the fieldwork, I wrote real-time notes on the laptop, which was always on hand except for in the ward milieu to preserve the patients’ sense of privacy and security. During the fieldwork, I spent most of my time in the nursing station and the staff break room in order to follow the daily conversations and take in information that was relevant for the inquiry. In total, I spent 146 h on 34 occasions at the unit during a period of 9 months. I did not participate in medical rounds or appraisals/employer calls. In total, the data comprised field notes (93 pages), discussions and group interviews (19 meetings). The 19 meetings were audio recorded and resulted in 548 min (about 9 h), equaling 463 text pages.

### Data Analysis

The data analysis cannot be explicitly extracted using more traditional and sequential methodologies. The different data analysis steps are therefore presented in the result section.

## Results

The research project took place between October, 2015, and June, 2016. According to the PAR methodology, the process itself is a part of the result and is therefore described in this section, starting with the problem identification phase and the planning ahead, the *prologue*. Next, we determined the actions and the process forward. This working phase included reciprocity between acting and reflecting (Springett et al. 2005), the *interlude*. In the later phase of the project, a working model called the *architecture of stability* was established and further visualized on a laminated card. And lastly, the project was evaluated, *the epilogue*. An overview of the process is shown in Table 1.

**Table 1:** Overview of the research process

Prologue	Interlude				Epilogue
Problem identification and planning	Action A	Action B	Reflecting	Action C	Reflecting
**Group discussions: identifying problems**	**Operationalization**	**Scrutinizing the rules**	**Linguistic transitions**	**Establishment of principles**	**Developing the ethics of stability**
There were numerous rules and routines	Identification of the 107 rules	Completing the questionnaire	Turning rules into principles	Safety	Ethics of deontology
There were unwritten rules and staff did not know how to deal with them		Group discussions		Structure	Ethics of consequentialism
There were written rules but some were open for interpretation	**Structural analysis**			Interplay	Ethics of care
Some staff requested more specific “how-to” rules	Written/unwritten				
Inconsistent application of rules risked negative consequences for the patients’ care	Internal/external				**Developing the work model**
Lack of consistency could place those who strictly followed rules in troublesome situations	Staff directed/patient directed				Architecture of stability
Uniformity of rules was associated with drawbacks for the patients	Interactive/administrative				
Individuals’ decisions risked criticism by colleagues	Flexible/rigid				**Evaluating the project**
Specific rules tended to become myopic to the extreme					
A rule-driven ward culture risked the establishment of even more rules					

### Prologue: Problem Identification and Planning Ahead

In accordance with PAR methodology, democratic principles were followed in the sense that the staff were collaborators as the research focus was identified. All staff were invited to a workplace meeting scheduled for a full day at a conference retreat location. Thereafter, the staff were divided into four groups made up of four to five members of staff with a mix of registered nurses and psychiatric aides. The groups were instructed to identify existing problems at the unit through brainstorming. This resulted in a prominent problem being identified by all groups and summarized as “ambiguity and doubts regarding ward rules”.

The staff acknowledged the existence of both written and unwritten rules. Two problems with written rules were that there were too many of them, and that they were open to interpretation and therefore perceived as ‘unclear’. Unwritten rules were additionally associated with ambiguity since no guidelines could support or justify these practices. The staff acknowledged that the ambiguity of how rules were put into practice fueled discord and conflicts within the work group. Some staff argued for the benefits of having clear-cut “*how to*”*-*rules that gave directions and structure in their daily work.

*It gets really difficult considering the fact that there are 25, 27 of us that work here and we might also think differently in many instances so that there should be some sort of routines, rules that help us in our work*. (Fathma, psychiatric aide)

*A structure that helps us carry out our work and that gives us something to fall back on. Because if we’re always reinventing the wheel in every new situation we get into, things get really tough*. (Sebastian, nurse)

The staff stated that uncertainty about how to put the rules into practice was not only an isolated work group-related problem; in addition they argued that inconsistent applications of rules risked negative consequences in the patients’ care and could by extension increase the length of hospital stays. Furthermore, if rules were communicated to patients and practiced differently due to staff’s individual assessments and decisions, it was believed by some staff members that it might place those who strictly followed the rules in troublesome situations. This phenomenon was explained as follows: when patients were given inconsistent directives, they were more likely to become frustrated and therefore posed a higher risk of acting out with threats and violence. It was argued that consistently implementing the rules with all patients would provide patients with a clearer structure. However, opposing arguments were also emphasized: uniformity was associated with drawbacks for the patients and their individual needs in specific situations. One staff told of a situation when she made an individual assessment and departed from the rule:

*[…] the exception for going outside and smoking. Because I did that here, I had a deal with a patient who had a ton of anxiety, and I let her go out onto the balcony when she wasn’t allowed to. And I got an earful for that, even though I told my manager about it and that I had an agreement with my patient*. (Lisa, psychiatric aide)

Those who argued for a more flexible style expressed concern that diverging from the regulations posed the risk of being criticized by colleagues. Another staff stated, “you can get sighs or looks that mean that they don’t think it’s okay” (Sarah, nurse). Getting *looks* from colleagues was brought up on several occasions during the problem identification phase. Conny, also a nurse, had had similar experiences: “Yes, I know, but you can get looks that say, ‘What are you doing?’ I’ve gotten that […] but, I mean, it’s not fun to get those kinds of looks.” One staff member who expressed a positive view of a more flexible approach stressed that rules tended to become myopic to the extreme:

*That honey should be given out for sore throats but there were many people that thought honey tasted good and it was like, no, it’s too expensive so it should be locked in the medication room and only be given out when a doctor ordered it… and then we were like, this rule was broken then and then there was a lot of discussion for a period of time. And that, you can’t, like, have a rule about honey, no, do you understand what I mean? It’s like, we can’t write 20 million rules on what we are allowed or not allowed to do. We need a framework that’s reasonable that you can work from, for example, safety, in other words the kinds of things that we can’t be flexible with. …* (Camilla, nurse)

The “honey rule” was one among the many *how-to* rules. Many of the staff advocated for omitting these kinds of rules and emphasized that rigidity and small-scope rules lead to a myopic and blurry perspective of the whole. Rather, it was argued that, as professionals, they should be able to make individual assessments and decisions based on the specific situation and patient. With a rule-driven ward culture, there was a tendency to establish even more rules: “It’s like this, that for every new patient, there’s a risk that we’ll make new rules or routines of some sort.” Even though opposing views existed and were acknowledged within the whole work group, there was a near-consensus that overly strict rules risked irritability among patients, which was not desirable; likewise, rules that were too loose could be a safety risk. Some staff members expressed concerns about rules and associated them with feelings of unease, since implementing rules often involved denying and/or restricting patients. Though opposing opinions were expressed, the staff consistently articulated the same end goal: they wanted a course of action that was “in the best interest of the patients.”

### Interlude: Planning and Determining the Action

The staff was again divided into four groups to brainstorm what kinds of actions could be taken to intervene. Afterwards, all four groups met in a large group and presented a summary of their discussions. It was decided that a two-step action plan for the research project should be implemented: (a) an operationalization and review of all rules and (b) based on the first action, the rules should be streamlined, and a core set of rules should be created as working guidelines for the staff. As one nurse put it, “just so that you know what applies” (Camilla, psychiatric aide); another staff said “we can’t have all these rules that manage every little detail.” (Carl, nurse).

#### Action A: Operationalization and Structural Analysis

After the problem identification phase, the fieldwork and collection of specific data began. The first step was to make an inventory of all the rules, using Spradley’s (1980) systematic way of observing and documenting artifacts. The guiding question was “What are all the rules in the unit?” In order to fulfill the task, I looked at the ward milieu and read folders, meeting notes, protocols and notices. For example, reminder notes were identified on the walls, in the WC and on the fridge. Throughout this process, the staff provided me with documents to ensure that all rules were included. From fieldwork observations it was possible to further add to the written rules with the unwritten rules. One example of an unwritten rule was that it was not allowed for patients to lie down on the floor of the ward; the staff said that the consequence of this was that they had to lift the patient off the floor and thereafter escort the patient to his or her room. Each rule was filed in separate rows in an Excel sheet; in total, 146 rules were identified. An in-depth review of the rules was conducted, and several of the rules were found to be almost identical. Hence, a condensation of the original 146 resulted in 107 rules. These were moved to a separate Excel sheet for further analysis.

Next, a structured analysis of the ward rules was conducted (Spradley 1980). The analysis aimed to examine the attributes (qualities or characteristics) of the identified rules; the objective of this analytical step was to find descriptive qualities for each rule. Spradley instructs the analyst to ask questions of the data. Three questions further guided the analysis: “What is the attribute?”, “In what ways do the rules differ from each other?” and “How do the rules relate to each other?” During this process, the identified attributes were discussed with the staff. In total, ten attributes were found and verified in discussion with staff. Each of the attributes was paired with a word that had the opposite meaning: written/unwritten, internal/external, staff-directed/patient-directed, interactive/administrative, flexible/rigid.

#### Action B: Scrutinizing and Condensing Rules

Again, the staff gathered in a conference facility outside the hospital for a full day to specifically work with ward rules. I was given the task of constructing a survey to scrutinize each rule. The survey asked if each rule should be left as is, re-phrased or omitted, and it asked participants to describe the intention of each rule. The purpose of the questionnaire was twofold: to rate each rule, and to function as a foundation for the group discussion that followed. The whole questionnaire was completed in about 90–120 min. Twenty-one staff members completed the poll, and about a third of the rules were recommended to be omitted, one-third, re-phrased and one-third, left as is. The main argument given for re-phrasing a rule was that it should not be stated that “it is not permitted to talk on mobile phones in the ward…” (John, psychiatric aide), but instead be re-phrased to state what is allowed, “you can use your mobile phone, but please make your private calls in your room.” (John, psychiatric aide). With regards to the safety rules, staff expressed consensus on the opinion that it was not possible to be flexible, and stressed the need to be consistent in the application of safety rules. However, due to the process of self-reflexivity throughout this project, the staff came to an awareness that the different ways of responding to patients’ requests were able to impact and mitigate the risk of patients becoming agitated.

#### Reflecting on Actions A and B: The Linguistic Transition

After completing the questionnaire, the staff was divided into four groups made up of four to five staff members, with a mix of registered nurses and psychiatric aides. They were instructed to discuss some of the 107 rules that they found most relevant for their work. All group discussions were audio recorded, and the sessions were limited to 60 min. Next, all four groups met in a large group. The groups presented, one by one, a brief summary of their internal discussions and the presentation was followed by a collective group discussion. During this large-group discussion, I took the role of a seminar leader. The large-group discussion was also audio recorded.

Throughout the discussions, staff expressed different reactions: “One thought was just how much power we have! We can make decisions about every aspect of a patient, including when they’ll use the toilet!” (Mona, nurse). Another said, “Wow, I see, there’s a rule about this, and I feel like, wow, I don’t know anything about that!” (Robert, psychiatric aide). It was apparent that the staff had different opinions regarding rules, but constructive discussions and reflection were necessary to enhance the awareness of rules and their impact on their work. In order to transcend the myopic perspective of the *how-to* rules, a change in the use of language was suggested. One staff member said, “Many of these rules are more like recommendations, that it’s not a rule that’s written in stone. So I don’t know if it’s a rule—but it’s a recommendation” (Carl, nurse), and right after, Sanja (psychiatric aid) added, “It makes these rules toothless. Then it’s better if you just call it a recommendation.”. Another suggestion for improvement was that the terminology should be changed, and the word ‘rule’ should be replaced by ‘principle’:

*… we shouldn’t have rules; instead we should have principles because principles cover so much more than rules do. Rules are… you’re not allowed to take this, and this, and this, and this—but the principle is that we’re not allowed to take things into the ward that patients can injure themselves with…* (Lars, nurse)

The linguistic transition of turning rules into principles was expressed as desirable and received a positive response in the large work group. Other staff members agreed and stressed that rules were not only fixed by nature; rules were also associated with negative consequences or punishments when they are violated.

*[…] as long as they’re called rules, what happens to me when I break a rule? That’s why I want to move away from the thinking that they’re rules. If you break a rule, it’s usually followed with a punishment, so to speak*. (August, psychiatric aide)

*When you work for a few years in a ward, you recognize all these rules, but it takes a pretty long time […] and that slap on the hand you get when you break a rule…* (Victor, nurse)

The question of consequences regarded both staff and patients. Staff stressed that the underlying idea of having rules and the negative consequences when breaking rules were not desirable, as it was seen as contradicting their view of professionalism in psychiatric care. In addition, negative consequences were seen as unsupportive when it came achieving a pleasant work climate. Hence, the term *principles* was argued to be broader, and staff would then have a greater possibility to make individual decisions due to specific situations.

#### Action C: Establishing a Set of Principles

The start of a linguistic transition was welcomed, although it was seen as necessary to pair the transition with something more concrete, as it otherwise would risk getting lost under more specific demands. This led to a realization that yet another action was needed. The unexpected events and moves back and forth in the different phases are typical for the processes in the PAR methodology. The next action was to establish a set of principles. In collaboration with the staff, the continued work involved taking the list of 107 rules as a point of departure, including the attributes from the structural analysis and the result from the questionnaire. One question guided the establishment of principles: “What do we want to achieve from a principle?” The discussions that followed emphasized that it was important to keep the unit safe while, at the same time, there was a desire to move away from the strict regulations and disciplining of the patients, but also to address the central aspects of how to approach patients with respect and individuality. Ideas, notes and emerging concepts and their correlations were drawn on a whiteboard, and then we discussed, revised some concepts and discussed further. We came to an agreement that three principles would include all aspects: 1), Safety, 2) Structure, and 3) Interplay.

### Reflecting: Development of the Principles of Intensive Psychiatry

Many staff held the view that they wanted to achieve a solution or a working model with instructions for *how to* act in specific situations. Contradicting this view, on numerous occasions staff simultaneously stressed that rules must be individualized, and expressed a desire to overcome the rules. Thus, when staff were not bound by rigid rules, it could potentially mitigate risks at work. Hence, the paradox of ward rules needed further analysis. The next sections add the dimensions of normative and applied ethical theories to the set of principles. The staff were used to discussing ethical matters in relation to their work in the psychiatric intensive care unit during monthly scheduled ethics rounds led by two hospital priests. During the project, I attended the ethics rounds and participated in the discussions. In addition to this, at several meetings and planning days, ethical matters were scheduled on the agenda.

#### The Ethics of Duty and the Interconnection to the Principle of Safety

Ethical discussions often addressed deontological ethics. First and foremost, Kant’s (2002) categorical imperative guides an action to be morally right, provided that you “act only in accordance with that maxim through which you can at the same time will that it become a universal law” (Kant 2002, p. 421). One maxim that the staff agreed to elevate to a universal law was to ensure safety. For example, a patient might become offended when denied a razor in the unit; however, according to Kant’s categorical imperative, the maxim of universal law outweighs the consequences. One staff said: “No, because the consequence here might be that the patient gets irritated, but it’s still right.” Safety was associated with a high degree of rigidity and low (or no) level of flexibility, no matter the consequences. The strong correlation was exemplified in a quote: “Yes, because… open a door, that you can actually do different things and make your own decision, but on the other hand you can’t give a knife to a patient because you want to be flexible.” The statement was followed by a general laughter, which signaled the uniformity of the attitudes towards safety aspects at work. In this sense, it is possible to morally justify actions due to the underpinning doctrine of deontology. Hence, the first principle was called ‘The Principle of Safety’ (also see Table 2).

**Table 2:** The architecture of stability

Principle of safety	Principle of structure	Principle of interplay
**Mona** Our duty is to help our patients based on the problems they have **Carl** We intervene in order to not allow injuries **Researcher** Duty… he says this, Kant, that you should act according to the maxim that could be held as a universal law **Mona** But can’t we say that we have a higher moral responsibility to not take in sharp knives. Because I think that’s really difficult, I mean that gets the brain going, can we defend something? **Sanja** But we staff members are allowed to use bread knives, scissors, everything so that it’s all right there, so that it’s just certain people in the ward that are allowed to use sharp objects then. But our duty is to make sure that no one who will abuse sharp things has access to them **Mona** And then there’s an assessment, should we assess each patient? Is it a patient that we know, that, yes, but this patient, the woman in the wheelchair, she’ll never abuse a knife, should she be allowed to get a knife? **Carl** No. If there’s anything that should be able to elevated to a universal law, it’s sharp objects out in common areas **Sebastian** Yes, that one can be elevated **Mona** That can certainly be elevated, yes **Carl** And then included in this is knowing what [which sharp objects] are there, can be there and aren’t there, will be in the ward if you say so, it’s like… and that you follow that and think that it really becomes purely ethical **Sebastian** But can’t we conclude that our duty here is to, to keep the ward safe? I mean, then all of what it means with that it should be safe, you know what I mean?	**Jonas** It’s also about other patients shouldn’t get scared by one who is acting out and such. Other patients shouldn’t feel bad and unsafe, and they really do sometimes **Hans** On the PICU there’s the ethics of consequences all the time. We expect patients will feel better because of something we do, and that’s the ethics of consequences, for example coercive measures… **Sanja** And separating is absolutely and directly the ethic of consequence **Sebastian** Yes **Carl** … so that the overall ward can tolerate it and feel happiness or enjoyment so, so… we need to take one person out of the environment **Jonas** Yes, exactly **Karin** That was a good example **Sebastian** Uh-huh **Camilla** A consequence is really stability **Researcher** A consequence is stability, Camilla says here **Camilla** Yes. That’s a place, we want to reach that **Jonas** But it can also be that the consequence [of a coercive measure] is maybe a violation in the moment but the limit can also be that this creates stability in the ward overall	**Kenneth** There’s no right or wrong, so you can never say, you should immediately do this or that, it depends on which patient it is, do we have someone we know, is it a returning patient […], he’s returning, someone we know very well and we know all these things, in that case maybe you do things in another way **John** … and it becomes another relationship with them [that we know] uh… and how we treat those patients in contrast with those that have been in only one time or many only, yes, some rather new patient that we don’t know so well… uh… it’s most often the patient that you don’t know so well that’s more at risk for harder limits and with the patient that your know well, then you can allow more based on the perspective that you know the patient better **Sanja** Or the opposite **Mona**: I was in in the morning and so she said this,”Oh, can you cut my hair, can you cut my hair, I’m so, my bangs are in the way and I have to get a haircut no, right this minute””No but…”, I said,”do you remember when you left here last time? … you thanked me because we hadn’t helped you to cut your hair, do you remember that?””No, no, no, no I don’t remember that.” Then afterwards… she thanked us because we had set limits with her **Jonas** I was thinking that the contact person, for example, has a very large role in this, to get to feel special and singled out and a part of their care **Sebastian** We have many patients what have been in for many years [on different care occasions] that you really have a special relationship with and that you have become known for your care role eventually

#### The Ethics of Consequentialism and the Interconnection to the Principle of Structure

Even though staff acknowledged that they wanted to change the numerous specific rules, it was at the same time consensus within the work group that a certain level of structure was needed. They stated that coercive measures were sometimes necessary to manage patients’ externalized symptoms, such as agitation, threats, and aggression towards other patients. Since patients affected each other within the closed ward environment, there was an imminent risk that one patient’s agitation could spread to and distort other patients’ behaviors, and the stability in the unit was then threatened. In order to maintain stability, the staff used of consequentialism to justify interventions, such as seclusion, as morally right—even though they violated the individual patient’s autonomy. Consequentialism is the group of normative ethical theories holding that the consequences of one’s conduct are the ultimate basis for any judgment about the rightness or wrongness of that conduct (Ellis 2014). Thus, a morally right act is one that will produce a positive outcome, or consequence. One staff said: “If a person is so sick that he or she can’t take responsibility for his or her own care, then we have to take responsibility.” (Karin, nurse). The imperative from this kind of reasoning was grounded in ‘for the greater good.’ The staff’s reasoning was in accordance with consequentialism, which is in conflict with the deontological ethics. Staff narrated different situations when they had applied consequentialist thinking. In one example, a patient had a limited number of cigarettes, and in the quote below a staff member paraphrased a conversation about structure and consequences:

*Yeah but now we have only a limited number, can we agree that you only smoke once an hour so that you’ll have enough cigarettes to last. No. And then you try to describe the consequences, then it will be like this, then you stay up, you wake up everyone else because you yell and scream because you don’t have any cigarettes. There are consequences if you don’t limit to once an hour*. (Lina, psychiatric aide).

### Ethics of Care and the Principle of Interplay

Although deontology and consequentialism provided good guidance in several situations to morally justify actions, both these ethical theories were on other occasions too restrictive. Feminist researchers have criticized ethical theories for their modernist assumptions. The main critics state that ethical theories presuppose the person to be impartial, while the ethics of care emphasizes the importance of response (Blum 2016; Gilligan 1982). However, when one is engaged in interpersonal relationships, which are complex, subjectivity must be involved. Hence, relational aspects must be considered due to flexibility and interplay in moral reasoning. Throughout the project, it was stated that different members of staff acted differently from one another; some members were more flexible, while other were more rigid. Throughout the many discussions, the main recurring phrase was “*it depends…*” which signaled a need for staff to be partial in their decisions. Staff acknowledged that they acted differently because they were different people, and they acted differently in relation to different patients in different situations. Sometimes rules could be bent on the basis that they *knew the patient* and had well-established relationships. However, well-established relationships and knowledge of a patient did not give immediate benefits for a patient so that they had ‘reached a higher status’ in the view of the staff—rather the opposite. An example by Mona is presented in Table 2.

### Epilogue

During the spring of 2016, the project was nearing completion. The project had been running for many months, and the topic had been discussed in numerous meetings. Also, we agreed that the summer vacation period at the end of June would be a natural point of closure. The last phase included the completion of the working model that could be visualized on a laminated card and an evaluation.

#### Research Project Outcome—The Architecture of Stability

**Fig. 1:**
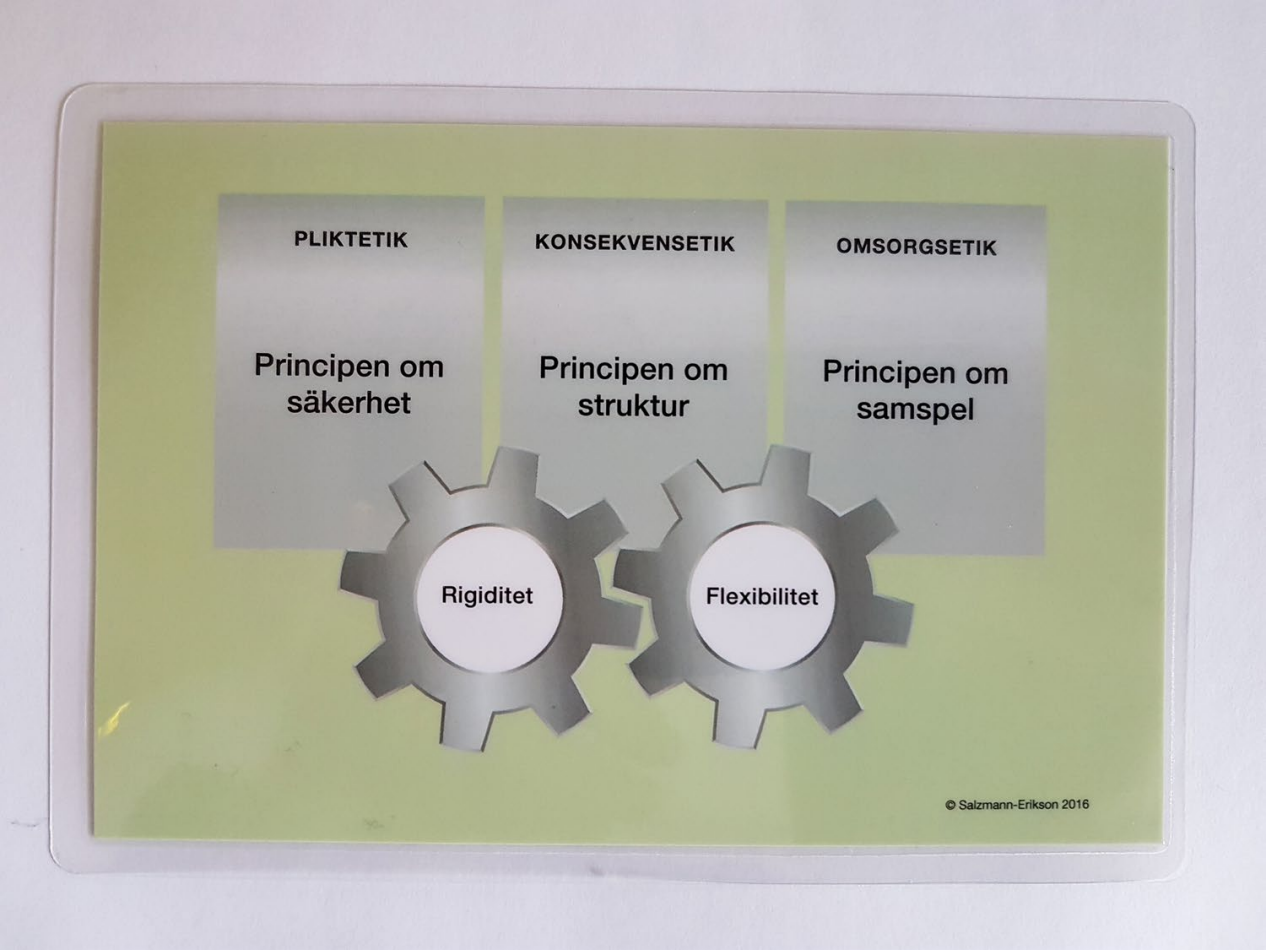

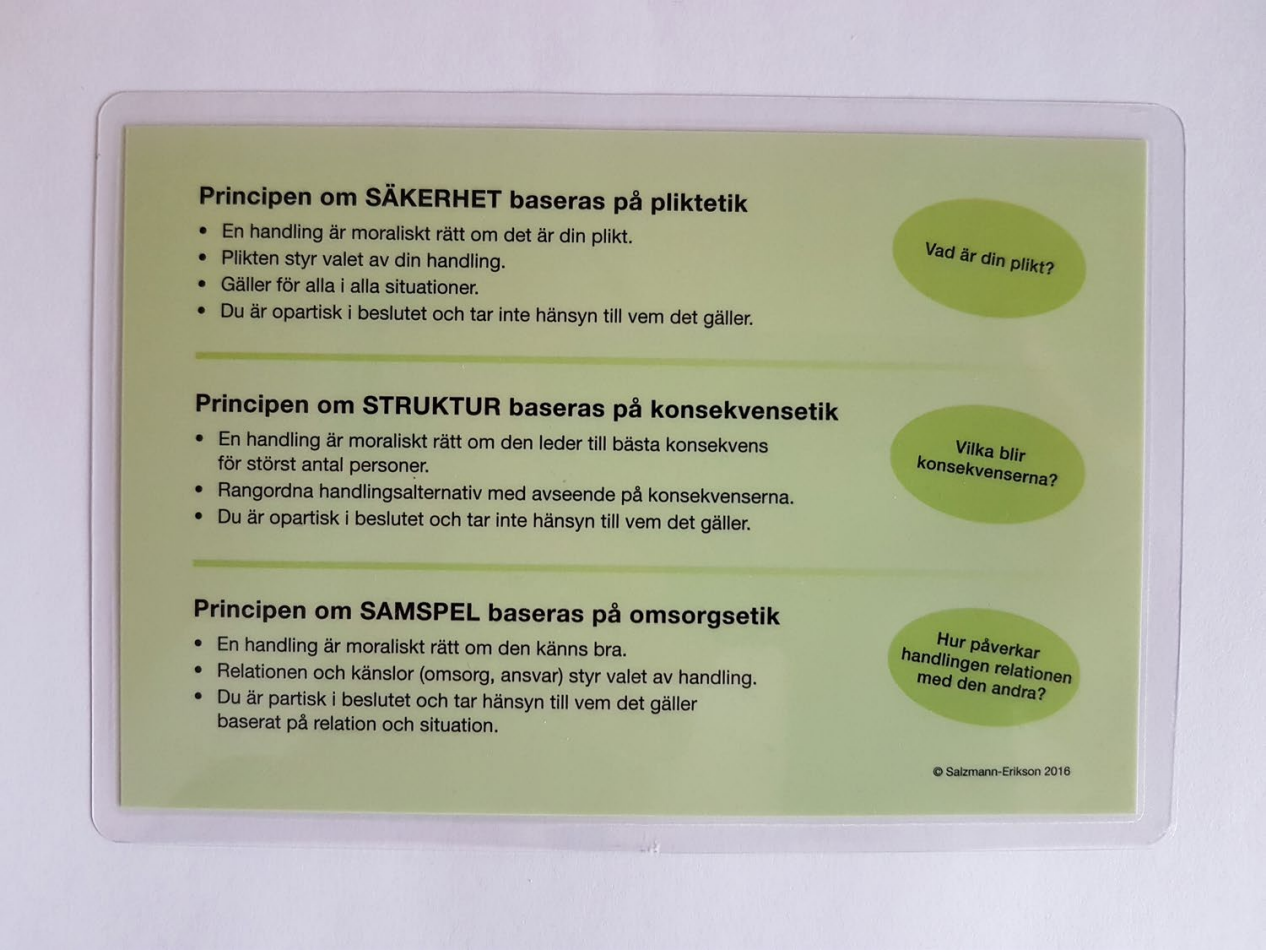

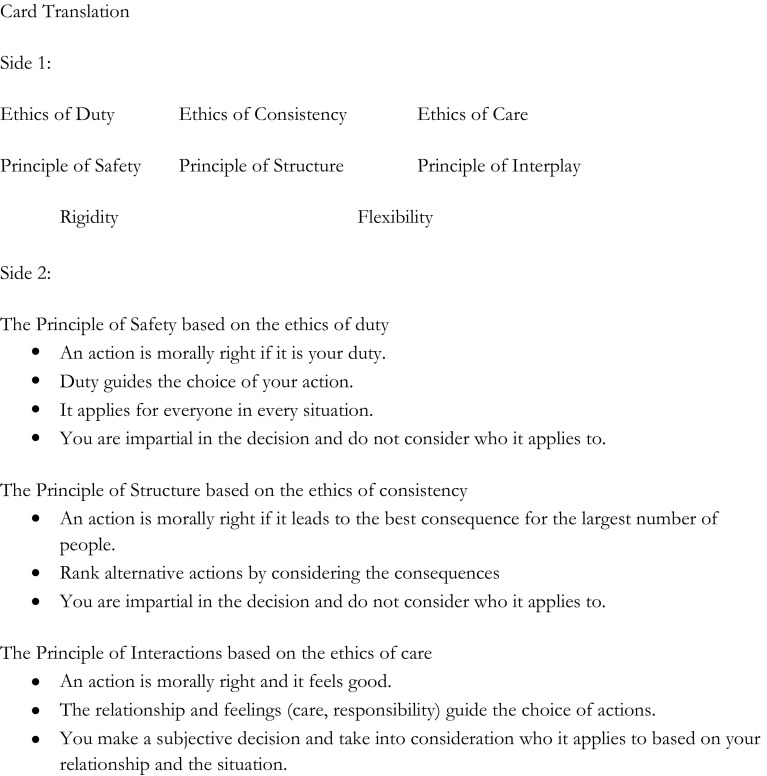
Image of the working model printed on a card

One aim of the project was to develop a working model to empower staff in their daily in-patient psychiatric nursing practices. Two concrete outcomes were requested since that would have more lasting effects. First, an abstract in Swedish was written in non-academic language that could be understood by all staff and new employees. Second, a card was produced. In collaboration with two staff members, a schematic figure was drawn to highlight the various aspects of the principles and the relationships to ethical schools of thought, while also illustrating the central concepts of rigidity and flexibility. We agreed that a laminated card should be produced, small enough to fit in the pocket of the nursing uniform. A graphic artist was consulted and given the task of creating an illustration of the working model and also designing the card. A draft from the graphic artist was emailed to the whole staff, inviting them to respond and make comments on the illustration, and some revisions were made. The card is presented as a photograph of the front and back in Fig. 1.

#### Evaluating the Project

The first part of the aim, to observe and analyze the process of staff development, was achieved. In its final phase, the project was orally evaluated. Overall, the staff were very satisfied and stated that taking part in the process itself had been a payoff, particularly the numerous reflection and discussion sessions. One nurse stated that staff members who had previously been rigid had begun to hesitate and become uncertain in situations when they previously had just ‘done what you should’. The doubt was considered important for thinking along new paths and was a sign of development towards a more complex moral awareness. Furthermore, it was stated that the level of flexibility and acceptance for individual assessments and decisions had been improved. Hence, the previous focus of consistency in the application of rules was transcended.

*I think we’ve gotten much, much better at respecting each other’s perspectives, we have different ways of relating to each other, it wasn’t long ago at all that you would almost bully a person, a caregiver. Because it’s not like that today*. (Kenneth, psychiatric aide)

Only a few staff members expressed negative feedback and that was regarding the length of the project.

It was stated that the working model, especially printed on a card, would constantly remind staff about the principles, empower their professionality, decrease collegial conflicts with increased acceptance for individual decisions, and in general, improve well-being at work.

## Discussion

In the process of this study, staff found that there were more than one hundred ward rules. These were scrutinized, and in this process, the word *rules* was relinquished in favor of adopting the term *principles*. No earlier studies have specifically accounted for the number of ward rules, so that result cannot be compared. However, the current study confirmed earlier study results that have highlighted ward rules as highly ingrained within the culture of in-patient psychiatry and used on a daily basis (Duxbury and Whittington 2005; Vatne and Holme 2006). The intention of applying ward rules in psychiatric units is clearly grounded from ‘safety thinking’ (Delaney and Johnson 2006; Salzmann-Erikson 2014). Even though several rules are clearly associated with safety, such as restricting patients from accessing sharp items, safety rationalizations risk becoming cliché, losing their meaning. Hence, as demonstrated in this study, psychiatric wards need to take a critical perspective on nursing practices and look above and beyond the limited aspects of safety. As mentioned in the Introduction, rules are disciplining rather than caring (Hall 2004; Morrison 1990; Watters 2000). In addition, Crichton (1997) states that rules bind both staff and patients “to behave.” That postulate can be used to understand the work group problems that were identified in the problem identification phase—some staff reported that they received unpleasant looks from colleagues when they bent or broke ward rules. Further, it is interesting to discuss what happens when rules are broken. Salzmann-Erikson (2013) stated that psychiatric nurses who broke away from their traditional roles and from other staff members’ expectations of them as professionals put themselves at risk of being criticized by colleagues. But stretching boundaries was considered by patients as highly important in their recovery process. The results in the present study underscore the value of *unbinding* (see Crichton 1997) staff from behaving in a predetermined manner and instead developing collegial tolerance towards flexibility and creativity in care. Hence, the staff will advance from passive clichés and executors of the institution and become self-reliant mental health professionals.

### Strengths and Limitations

In this participatory action research project, I have observed and analyzed the process of a team development project in a psychiatric intensive care unit. The point of origin of the project was to unconditionally engage myself as a researcher in a work group with the intention of improving working conditions and empowering their professional abilities. PAR methodology was well-suited for this project. The non-authoritative approach was necessary to identify issues or areas of improvement that felt meaningful for the work group. The allowance for time, resources and engagement from the work group made it possible to take the issues to the table. Over a long period of time, processing, discussing and being self-critical made it possible to progress and develop the new working model. One major limitation of this study was that patients were not included in the data collection. Video recordings of situations and interactions and processes of negotiations between patients and nurses when ward rules were applied would have given highly interesting data to analyze and would have developed the working model further. The Ethical Review Board did not consent to that kind of data collection; hence it was not possible to study interactions and processes of negotiation between patients and nurses. Since the staff was not observed after starting to work with the new model, the ways in which they may use the model to mitigate risks at work is restricted to the section “Evaluating the project” The application of the new model and how it empowers staff and mitigate risks from various variables is appropriate for a future research project.

### Reflexivity

Gaining trustworthiness when using the PAR methodology is especially important and correspond to the approach of ‘researcher-as-instrument’; thus, this calls for reflexivity and self-reflexivity (Bergold and Thomas 2012; Morrow 2005). Throughout this project, I wrote about the research process, the people I met, my observations, impressions, thoughts and feelings. I tried to pay attention to statements which were in line with my own philosophy, but it was just as important to reflect upon statements which contrasted my own opinions. Throughout the process, my strategy was to ask follow-up questions rather than confirm or disconfirm the staff. Moreover, other PAR methodologists have emphasized researchers’ reflexivity, not only as a quality criterion but also to equalize power dynamics between the researcher and the participants (Gatenby and Humpries 2000; Maguire 2006). During this project, I constantly reflected on my own influence on authority and power dynamics and how it affected the relationship, and how I might approach staff in order to flatten power. For example, during a general nurse meeting my plan was to add a subject to the meeting agenda, “Discuss written ward rules”. Due to heavy workload and exhaustionI sensed an atmosphere of down-heartedness in the meeting room and an aversion to discuss anything. From my philosophy of researcher ethics I did not push forth my demand for a discussion. Instead, I decided to not audio record the meeting and I adopted a passive stance and joined the silence over a cup of coffee. Over the course of the project my role took different shapes. Sometimes, I was a professional researcher using academic language to inform about the study and reconciliations about the progress. In more everyday occasions, I adopted a more relaxed role and engaged in private discussions. The ability for me to adopt different roles in certain situations was a technique that I developed during previous fieldwork in other studies.

### Clinical Implications

**Fig. 2:**
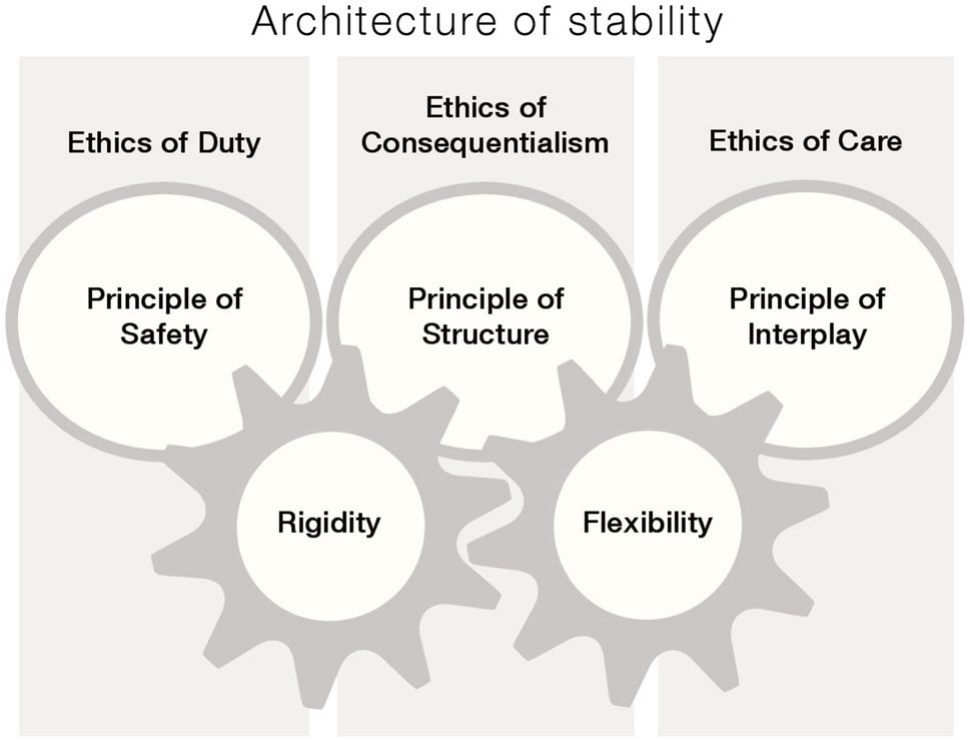
Visualization of the architecture of stability

A workplace culture is defined by the staff’s assumptions, values, norms and attitudes, and expressed in symbols, for example by the way staff members act and use language (cf. Janićijević 2012). This study challenges the habitual use of language in terms of the linguistic transition. The term *rules* was changed into the term *principles*; whereas the former was connotated to an institutional-power language, the latter invites discussion. Explicitly, the linguistic transition may be seen as minor, but as language reflects a culture’s norms and values, the transition in language has a major implication for the clinical practice as it mitigates risks. However, further research is needed to quantitatively measure the outcome of implementing this new working model. Furthermore, the visualization (Fig. 2) of the working model was concretized on a printed laminated card—*the Architecture of Stability*. The visualization has a profound incitement for clinical implications as it supports staff when they face complex situations, and provides guidance and arguments for decisions. Staff in psychiatric wards can make use of the working model, which includes the principles of safety, structure and interplay. The working model includes the important aspect of safety but is not restricted to that view. In order to deal with many individual patients, their problems and needs, as well as different colleagues in specific situations that occur unexpectedly, a working model has to acknowledge nursing practices on a larger scale. Staff cannot make decisions solely based on deontology and consequentialism but should also include a view that includes subjective and relational aspects. Henceforward, the pioneer contribution to clinical practice this study suggests is to acknowledge the *ethics of totality*.
